# Efficacy of Expressed Breast Milk Versus Sweet Solutions for Neonatal Procedural Pain: A Systematic Review and Meta-Analysis

**DOI:** 10.3390/biomedicines14071565

**Published:** 2026-07-13

**Authors:** Vanessa Espejo-Mansilla, Alessandra Coscia, Héctor Fuentes-Barría, Raúl Aguilera-Eguía, Lisse Angarita-Davila, Cherie Flores-Fernández, Ángel Roco-Videla

**Affiliations:** 1 Escuela de Obstetricia, Facultad de Medicina, Universidad Andres Bello, Concepción 3349001, Chile; v.espejomansilla@gmail.com; 2 Neonatology Unit, Department of Public Health and Pediatrics, University of Turin, 10124 Torino, Italy; alessandra.coscia@unito.it; 3 Centro de Investigación en Medicina de Altura (CEIMA), Universidad Arturo Prat, Iquique 1110939, Chile; 4 Departamento de Salud Pública, Facultad de Medicina, Universidad Católica de la Santísima Concepción, Concepción 3349001, Chile; raguilerae@ucsc.cl; 5 Escuela de Nutrición y Dietética, Facultad de Medicina, Universidad Andres Bello, Concepción 3349001, Chile; lisse.angarita@unab.cl; 6 Departamento de Gestión de la Información, Universidad Tecnológica Metropolitana, Santiago 7550000, Chile; cflores@utem.cl; 7 Dirección de Desarrollo y Postgrado, Universidad Autónoma de Chile, Santiago 7500912, Chile; angel.roco@uautonoma.cl

**Keywords:** infant, breast feeding, procedural pain, sweetening agents, analgesia

## Abstract

**Objective:** To evaluate the efficacy of expressed breast milk in reducing procedural pain in neonates compared with sweet solutions and to assess differences according to type of sweetener and timing of pain assessment. **Materials and Methods:** A systematic review and meta-analysis of randomized controlled trials was conducted following PRISMA guidelines and registered in PROSPERO (CRD420261418093). PubMed/MEDLINE, Scopus, CINAHL, Cochrane Library, and Google Scholar were searched through May 2026. Random-effects models using standardized mean differences (Hedges’ g) were applied. Heterogeneity was assessed using the I^2^ statistic. Meta-analyses were conducted by pain assessment time point, with subgroup and sensitivity analyses. Risk of bias was assessed using RoB 2 and certainty of evidence (CoE) using GRADE. **Results:** Seventeen randomized controlled trials were included. No statistically significant differences were observed between expressed breast milk and sweet solutions during the procedure (SMD = 0.15; 95% CI −0.18 to 0.47; I^2^ = 82.6%; Low CoE), at 1 min post-procedure (SMD = 0.40; 95% CI −0.11 to 0.92; I^2^ = 84.5%; Low CoE), or at 2 min post-procedure (SMD = 0.27; 95% CI −0.14 to 0.69; I^2^ = 7.9%; Moderate CoE). Subgroup analyses showed no significant differences or interactions. Egger’s test detected significant funnel plot asymmetry during the procedure (intercept = −3.61; 95% CI −5.53 to −1.69; *p* = 0.005), but not at 1 min post-procedure (intercept = 1.84; 95% CI −3.53 to 7.20; *p* = 0.521). **Conclusions:** Current evidence suggests that expressed breast milk provides analgesia comparable to sweet solutions for neonatal procedural pain, although the certainty of the evidence is limited by substantial heterogeneity.

## 1. Introduction

Neonatal pain is defined, according to the International Association for the Study of Pain (IASP), as an unpleasant sensory and emotional experience associated with actual or potential tissue damage [1]. In this context, for decades, it was assumed that newborns could not perceive pain due to the immaturity of the central nervous system and the presumed absence of long-term pain memory [2,3].

This belief led to the performance of multiple invasive procedures without adequate analgesia, including surgical interventions. However, since the 1980s, this view has been widely challenged, and it is now established that neonates possess functional nociceptive capacity from early stages of development [4,5]. Current evidence consistently shows that the anatomical and functional components of the nociceptive system are present from approximately 25–26 weeks of gestation, including peripheral, spinal, and supraspinal pathways necessary for pain transmission [4,6].

In the perinatal and neonatal context, exposure to nociceptive stimuli is frequent [7]. Newborns are routinely subjected to potentially painful procedures from birth, such as heel lances, venipunctures, and intramuscular administration of vitamin K [5,8]. In neonatal intensive care units, this exposure may reach a high number of daily procedures, often without consistent analgesic strategies [7]. Evidence suggests that repeated exposure to pain during this critical period of neurodevelopment is associated not only with acute physiological effects but also with long-term alterations in neurocognitive development, behavior, and stress regulation [9,10]. In addition, modifications in the hypothalamic–pituitary–adrenal axis, altered brain connectivity, structural changes in brain development, and epigenetic mechanisms related to early exposure to stress and pain have been described [11,12].

From the perspective of the maternal–infant axis, breastfeeding and early exposure to breast milk represent key components of the early postnatal nutritional environment, with potential impacts on neonatal physiological regulation [13,14]. Breast milk is a complex biological matrix containing multiple bioactive compounds, including lactose, tryptophan, opioid-like peptides, hormones, and other neuromodulatory mediators, which participate in the regulation of stress, behavior, and neonatal homeostasis [13,14,15]. Furthermore, sensory stimuli associated with its oral administration, such as taste, smell, and sucking, may activate neurobehavioral pathways involved in self-regulation and pain modulation [16].

In this sense, breast milk can be understood not only as a nutritional source but also as an early bioactive exposure with potential modulatory effects on neonatal neurophysiological responses. It has therefore been proposed as a non-pharmacological analgesic intervention due to its biochemical and sensory profile, as well as its potential interaction with endogenous pain modulation mechanisms [17,18]. However, higher-level evidence suggests that its analgesic effects are not uniform but rather context dependent. Evidence from umbrella reviews and meta-analytic reanalyzes, including Gök et al. [18], indicates that breastfeeding and breast milk-related sensory exposure can reduce behavioral and physiological indicators of procedural pain, with breastfeeding showing the most consistent and pronounced effects across studies. Similarly, Çamur et al. [19], in a systematic review and meta-analysis of randomized controlled trials, reported significant reductions in neonatal procedural pain with breastfeeding and breast milk-based interventions, including expressed breast milk, taste, and odor exposure, with overall medium-to-large effect sizes across behavioral and physiological outcomes. Together, these findings provide converging evidence that breast milk-based interventions are effective, but they also highlight substantial heterogeneity across populations, procedures, and intervention modalities, suggesting that their analgesic efficacy is strongly influenced by clinical context rather than representing a uniform pharmacological effect.

Considering the importance of maternal nutrition and breastfeeding as early determinants of neonatal development, it is relevant to synthesize the available evidence regarding the potential pain-modulating effect of expressed breast milk during painful procedures. Therefore, the aim of this systematic review and meta-analysis is to evaluate the efficacy of expressed breast milk in reducing procedural pain in neonates compared with sweet solutions and to assess differences according to type of sweetener and timing of pain assessment, contributing relevant evidence within the framework of maternal and infant nutrition in early life stages.

## 2. Materials and Methods

### 2.1. Design

The protocol was developed following the recommendations of the guidelines of the Preferred Reporting Items for Systematic Reviews and Meta-Analyses (PRISMA) (Supplementary Materials) [20]; approval code: PROSPERO CRD420261418093. The final implementation of the study deviated from the registered PROSPERO protocol with respect to the inclusion of sensitivity analyses and the assessment of the certainty of evidence using the GRADE approach.

### 2.2. Eligibility Criteria

Eligibility for study inclusion was determined using the P.I.C.O.S framework, which includes participants, intervention, comparison, outcome, and study design, with the following specifications:

- Population: Neonates (preterm and/or term) undergoing painful procedures.
- Intervention: Oral administration of expressed breast milk.
- Comparison: sweeteners as non-pharmacological analgesic strategies (sucrose, glucose, dextrose or others).
- Outcome: Pain assessed using validated neonatal pain scales.
- Study design: Randomized controlled trial.

### 2.3. Data Sources and Search

The literature search was conducted in the following electronic databases: PubMed/MEDLINE, Scopus, CINAHL, and the Cochrane Library, from their inception until 22 May 2026. Additionally, a supplementary search was performed in Google Scholar to identify potentially relevant studies not retrieved using the primary electronic search strategies.

Boolean operators AND and OR were used, employing MeSH (Medical Subject Headings) terms and free-text terms related to the study topic, such as “Breast milk,” “Expressed breast milk,” “Neonate,” “Newborn,” “Pain,” “Analgesia,” and “Procedural pain,” combined according to the defined search strategy in Table 1.

No language restrictions were applied during the database search. However, studies for which a full-text translation could not be obtained were excluded during the eligibility assessment.

### 2.4. Study Selection and Data Collection

Two reviewers independently screened the titles, abstracts, and full texts of all identified studies to assess eligibility. Any disagreements were resolved by a third reviewer, who acted as an arbitrator. Relevant data were extracted from each study, including first author, publication year, study population, interventions or comparisons, primary outcomes, and methodological characteristics. In cases of missing or unclear information, attempts were made to contact the corresponding author for clarification.

### 2.5. Methodological Quality Assessment

The methodological quality of the studies included was evaluated using the Risk of Bias 2 (RoB 2) tool developed by the Cochrane Collaboration [21]. This tool assesses and classifies potential biases in randomized controlled trials across five key domains: (D1) the random sequence generation process, (D2) deviations from assigned interventions, (D3) missing outcome data, (D4) outcome measurement, and (D5) selection of reported outcomes.

The risk of bias and quality of evidence were assessed independently by two researchers. Discrepancies regarding the inclusion of specific articles were resolved by a third reviewer, who acted as an arbitrator. This structured process allowed for clear and transparent conclusions regarding the quality of the evidence in the review.

### 2.6. Strategy for Data Synthesis

The quantitative synthesis was conducted using the online platform MetaAnalysisOnline (https://metaanalysisonline.com) [22]. A random-effects meta-analysis model was implemented to account for the expected clinical and methodological heterogeneity among the included studies. Effect sizes were estimated as standardized mean differences (SMDs) using Hedges’ g, allowing comparison across different neonatal pain scales while correcting for small-sample bias according to Cochrane methodology [23,24]. Between-study variance (τ^2^) was estimated using the restricted maximum likelihood (REML) method, and inverse-variance weighting was applied within the random-effects framework [25]. A Knapp–Hartung adjustment was used to obtain more accurate confidence intervals by accounting for uncertainty in the estimation of between-study variance, thereby reducing the risk of spurious statistical significance [26].

Statistical heterogeneity was assessed using Cochran’s Q test and quantified using the I^2^ statistic, with thresholds of 25%, 50%, and 75% representing low, moderate, and high heterogeneity, respectively [27].

The results of the platform MetaAnalysisOnline (https://metaanalysisonline.com) were presented using a forest plot format consistent with Cochrane’s RevMan software. [22].

### 2.7. Subgroup Analyses and Publication Bias

Subgroup analyses were performed according to the type of sweet comparator solution (glucose, dextrose, and sucrose) and according to the timing of pain assessment (during the procedure, 1 min after the procedure or intervention, and 2 min after the procedure or intervention). These analyses were planned to explore potential sources of clinical and methodological heterogeneity across the included studies. No additional subgroup analyses were performed to avoid unreliable interpretations due to the limited number of studies and potential loss of statistical power.

Publication bias was assessed using funnel plot visualization when at least 10 studies were available for a given outcome [28]. Meta-regression was not performed because fewer than ten studies were available within each subgroup and important covariates were inconsistently reported across trials, limiting the feasibility and validity of moderator analyses. According to the Cochrane Handbook for Systematic Reviews of Interventions, meta-regression should be interpreted with caution when the number of studies is small, as results may be unstable and prone to spurious associations [24].

### 2.8. Sensitivity Analysis

Sensitivity analysis to assess the robustness of the results included:

- Exclusion of studies classified as having a high risk of bias according to the RoB 2 tool, in order to explore the stability of the findings after removing methodologically weaker evidence [21].
- Use of a single effect estimate per study, prioritizing the pain assessment time point closest to the painful procedure, to avoid the potential influence of multiple measurements derived from the same trial.
- Sequential exclusion of each study (leave-one-out analysis) to evaluate the influence of individual studies on the overall pooled effect size and heterogeneity.

### 2.9. Certainty of Evidence

The certainty of the evidence for each outcome was assessed using the GRADE approach [28]. Evidence from randomized trials was initially considered to be of high certainty and was subsequently downgraded based on limitations related to risk of bias, inconsistency, indirectness, imprecision, and publication bias. The assessment was conducted independently for each clinically relevant outcome, including pain during the intervention, at one minute, and at two minutes post-intervention.

A complete and independent evaluation of the certainty of the evidence was performed for each outcome using the GRADE approach, which considers domains related to study design, risk of bias (assessed using RoB 2.0), inconsistency, indirect evidence, imprecision, publication bias, and other considerations [21,24,27,28]. The certainty of evidence was classified into four levels: high, moderate, low, and very low [29]. A partially contextualized approach was applied, using the null effect as the threshold to determine whether the interventions provided a clinically meaningful benefit compared with the comparator [29,30].

Conclusions were formulated following the standardized language proposed by the GRADE Working Group, combining effect size with the level of certainty [29,30]: high certainty (the intervention has an effect), moderate certainty (the intervention probably has an effect), low certainty (the intervention may have an effect), and very low certainty (the evidence about the effect is very uncertain). This approach facilitates clear communication of the findings, in line with current GRADE standards for systematic reviews [30].

## 3. Results

### 3.1. Study Selection

The bibliographic search identified a total of 1329 records, of which 1328 were retrieved through electronic databases (PubMed, Scopus, Web of Science, Embase, and Google Scholar) and one through other sources. Google Scholar was used as a supplementary search engine to broaden the search strategy and capture potentially relevant studies not indexed in traditional databases; however, consistent with methodological guidance from the Cochrane Handbook and previous evidence on search reproducibility, its use was restricted to an adjunct role due to its large yield of non-specific and duplicate records and limited transparency of search algorithms. Therefore, only records not already identified in the primary databases and meeting the predefined inclusion criteria proceeded to screening within the PRISMA workflow [24,31]. After the removal of 424 duplicate records, 905 records underwent title and abstract screening. Of these, 844 were excluded for not being related to the objective of the review based on the evaluation of titles, abstracts, and keywords.

Subsequently, 61 articles were considered potentially eligible for full-text assessment. However, five studies could not be retrieved [32,33,34,35,36]. Consequently, 56 full-text articles were assessed for eligibility [37,38,39,40,41,42,43,44,45,46,47,48,49,50,51,52,53,54,55,56,57,58,59,60,61,62,63,64,65,66,67,68,69,70,71,72,73,74,75,76,77,78,79,80,81,82,83,84,85,86,87,88,89,90,91].

**Figure 1:**
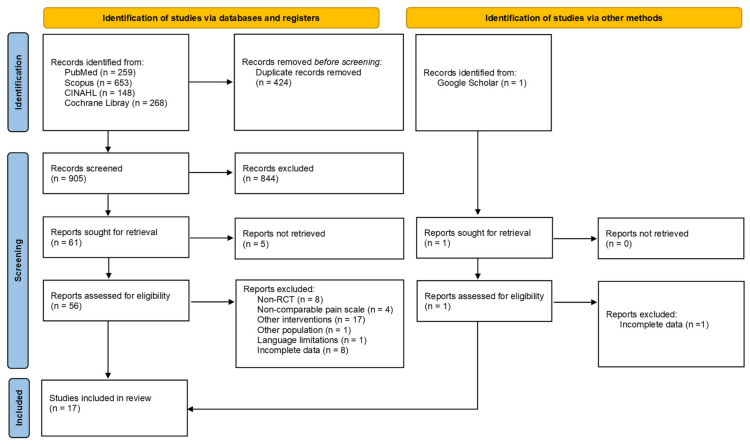
PRISMA flowchart for the search, identification, screening and selection of analyzed studies.

Among the full-text articles evaluated, eight studies were excluded because they were not RCTs [37,38,39,40,41,42,43], four due to the use of non-validated or non-comparable pain assessment scales [44,45,46,47,48], and seventeen because they evaluated interventions that did not meet the predefined inclusion criteria [49,50,51,52,53,54,55,56,57,58,59,60,61,62,63,64,65]. Additionally, one study was excluded for language limitations [66], and one because it did not directly assess a neonatal population [67]. Finally, eight studies were excluded from the quantitative synthesis because they did not provide sufficient data for inclusion in the meta-analysis [44,68,69,70,71,72,73]. Therefore, 17 studies were ultimately included in the meta-analysis (Figure 1) [74,75,76,77,78,79,80,81,82,83,84,85,86,87,88,89,90].

### 3.2. Characteristics of the Included Studies

Table 2 shows a total of 17 RCTs with parallel-group designs, published between 2006 and 2024, included in the quantitative synthesis, comprising a total of n = 1572 neonates.

The included studies were conducted across diverse geographic regions, with the largest contributions from India (n = 5) and Turkey (n = 3). Additional studies were conducted in Iran (n = 2), and single studies were identified from Italy, Brazil, Spain, South Korea, Pakistan, Taiwan, and the United States, reflecting a broad international interest in non-pharmacological interventions for neonatal procedural pain.

The study populations included both preterm and term neonates, with gestational ages ranging from extremely preterm infants (≤32 weeks) to full-term newborns (37–42 weeks). Most studies focused on preterm or late preterm neonates, particularly those between 30 and 37 weeks of gestation, a population frequently exposed to repeated painful procedures in Neonatal Intensive Care Units (NICUs).

Sample sizes varied widely, ranging from 33 to 400 neonates. The primary intervention across all studies was orally administered EBM, delivered mainly via syringe, although some studies used droppers, pacifiers, nipples, or paladai feeding devices. The administered volume ranged from 0.3 mL/kg to 5 mL, depending on gestational age, clinical context, and study protocol. In most trials, EBM was administered approximately 1–2 min before the painful procedure.

Painful procedures included heel lance, retinopathy of prematurity (ROP) screening, venipuncture, nasopharyngeal suctioning, and orogastric tube insertion. Heel lance was the most frequently assessed procedure, followed by ROP screening. Comparator interventions included oral sucrose (20–33%), glucose or dextrose solutions (10–25%), sterile water, or routine care.

Pain was assessed using validated neonatal pain scales. The Premature Infant Pain Profile (PIPP/PIPP-R) was the most frequently used instrument, followed by the Neonatal Infant Pain Scale (NIPS), Neonatal Pain, Agitation and Sedation Scale (N-PASS), Douleur Aiguë du Nouveauné (DAN), NIAPAS, and ALPS-Neo. Outcomes were assessed at heterogeneous time points, including during the procedure and within the first 0.5 to 15 min of post-intervention.

### 3.3. Risk of Bias in the Included Studies

**Figure 2:**
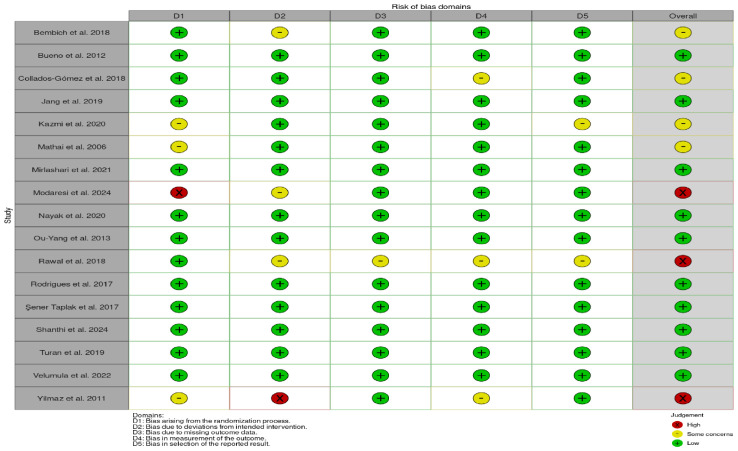
Risk of bias RoB 2 [21], in the studies included [74,75,76,77,78,79,80,81,82,83,84,85,86,87,88,89,90].

Figure 2 summarizes the risk-of-bias assessment performed using the RoB 2 tool across the five domains (D1–D5) [21]. Overall, most included studies were judged to have a low risk of bias, supporting the methodological robustness of the evidence base. Ten studies were classified as having an overall low risk of bias [75,77,80,82,83,85,86,87,88,89], four were judged as having some concerns [74,76,78,79], and three were rated as having an overall high risk of bias [81,84,90].

Regarding the randomization process (D1), one study was judged to be at high risk of bias [81], three studies raised some concerns [78,79,90], and the remaining thirteen studies were assessed as having a low risk of bias [74,75,76,77,80,82,83,84,85,86,87,88,89]. For deviations from intended interventions (D2), one study was rated as high risk of bias [90], three studies raised some concerns [74,81,84], and the remaining thirteen studies were judged to have a low risk of bias [75,76,77,78,79,80,82,83,85,86,87,88,89].

For missing outcome data (D3), all studies were assessed as having a low risk of bias except Rawal et al. [84], which raised some concerns. Regarding the measurement of the outcome (D4), three studies [76,84,90] were judged as having some concerns, whereas the remaining studies were rated as having a low risk of bias [74,75,77,78,79,80,81,82,83,85,86,87,88,89]. Finally, for the selection of the reported result (D5), two studies [78,84] raised some concerns, while all remaining studies were judged to have a low risk of bias [74,75,76,77,79,80,81,82,83,85,86,87,88,89,90].

### 3.4. Effect of Expressed Breast Milk Versus Sweet Solutions on Neonatal Pain During the Procedure

**Figure 3:**
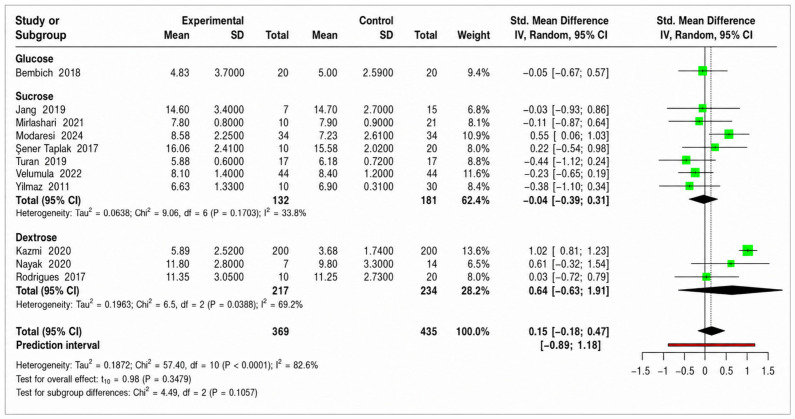
Effect of Expressed Breast Milk versus Sweet Solutions on Neonatal Pain During the Procedure in the studies included [74,77,78,80,81,82,85,86,88,89,90].

The meta-analysis of neonatal pain during the procedure showed a small and non-significant overall effect (SMD = 0.15; 95% CI −0.18 to 0.47; *p* = 0.35). The 95% prediction interval ranged from −0.89 to 1.18, indicating that the true effect in a future comparable study could favor either EBM or sweet solutions. The confidence interval crossed the null value, indicating no statistically significant difference between EBM and sweet solutions overall. Substantial between-study heterogeneity was observed (I^2^ = 82.6%), suggesting considerable variability in treatment effects across studies. Subgroup analyses demonstrated no statistically significant differences between EBM and either glucose/sucrose (SMD = −0.04; 95% CI −0.39 to 0.31; I^2^ = 33.8%) or dextrose (SMD = 0.64; 95% CI −0.63 to 1.91; I^2^ = 69.2%). Moreover, no significant differences were observed between subgroups (χ^2^ = 4.49, *p* = 0.11). Overall, the available evidence does not demonstrate a statistically significant difference between EBM and sweet solutions for reducing neonatal procedural pain during the intervention (Figure 3).

**Figure 4:**
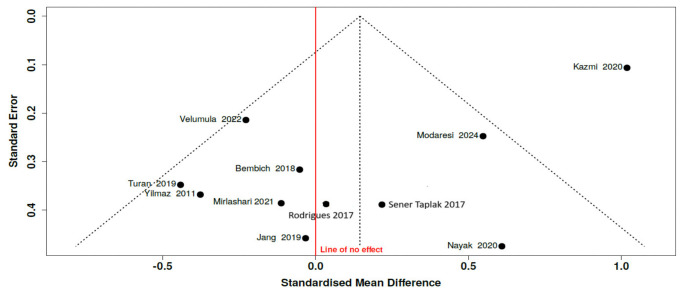
Funnel Plot on the Overall Effect of Expressed Breast Milk versus Sweet Solutions on Neonatal Pain During the Procedure in the studies included [74,77,78,80,81,82,85,86,88,89,90].

Visual inspection of the funnel plot (Figure 4) indicated potential publication bias. The Egger’s test supports the presence of funnel plot asymmetry (intercept: −3.61, 95% CI: −5.53 to −1.69, t: −3.692, *p* = 0.005).

Leave-one-out sensitivity analyses showed that sequential exclusion of individual studies did not materially alter the pooled effect estimate or its statistical significance, with SMDs ranging from 0.01 to 0.20. Across all leave-one-out analyses, the 95% prediction intervals consistently crossed the null value, indicating that the expected effect in a future comparable study could favor either EBM or sweet solutions despite the exclusion of individual studies. Exclusion of Kazmi et al. [78] substantially reduced heterogeneity (I^2^ decreased from 82.6% to 16.6%) and markedly narrowed the prediction interval (−0.52 to 0.53), although the overall effect remained non-significant (SMD = 0.01; 95% CI −0.25 to 0.26). These findings indicate that, although Kazmi et al. [78]. contributed importantly to between-study heterogeneity, the overall conclusions of the meta-analysis were robust (Table 3).

### 3.5. Effect of Expressed Breast Milk Versus Sweet Solutions on Neonatal Pain After 1 Minute Post-Procedure

The meta-analysis of neonatal pain after 1 min post-procedure showed a small and non-significant overall effect (SMD = 0.40; 95% CI −0.11 to 0.92; *p* = 0.37). The 95% prediction interval ranged from −1.16 to 1.96, indicating that the true effect in a future comparable study could favor either EBM or sweet solutions. The confidence interval crossed the null value, indicating no statistically significant difference between groups. Substantial between-study heterogeneity was observed (I^2^ = 84.5%), suggesting considerable variability in effect estimates across studies.

Subgroup analyses according to type of sweet solution also showed no statistically significant differences between groups for glucose-based solutions (SMD = 0.34; 95% CI −4.99 to 5.67; I^2^ = 81.9%), sucrose (SMD = 0.10; 95% CI −0.59 to 0.80; I^2^ = 76.6%), or dextrose (SMD = 0.94; 95% CI −1.42 to 3.29; I^2^ = 86%). In all cases, the wide confidence intervals crossing the null value indicate high imprecision and uncertainty in the subgroup-specific estimates.

**Figure 5:**
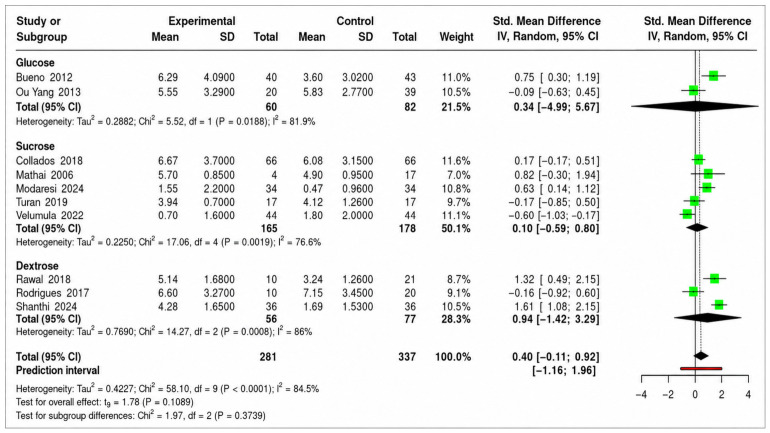
Effect of Expressed Breast Milk versus Sweet Solutions on Neonatal Pain After 1 Minute Post-procedure in the studies included [75,76,79,81,83,84,85,87,88,89].

Importantly, no statistically significant differences were observed between subgroups (χ^2^ = 1.97, *p* = 0.37), indicating that the type of sweet solution did not significantly modify the effect of the intervention on neonatal pain after 1 min post-procedure. Overall, the available evidence does not demonstrate a statistically significant difference between groups for reducing neonatal procedural pain after 1 min post-procedure (Figure 5).

**Figure 6:**
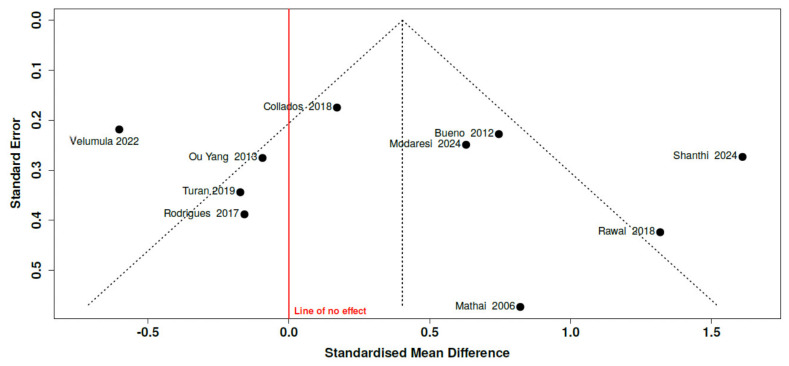
Funnel Plot on the Overall Effect of Expressed Breast Milk versus Sweet Solutions on Neonatal Pain After 1 Minute Post-procedure in the studies included [75,76,79,81,83,84,85,87,88,89].

Visual inspection of the funnel plot (Figure 6). Consistent with this interpretation, the funnel plot does not suggest substantial publication bias, and Egger’s regression test did not detect significant funnel plot asymmetry (intercept = 1.84, 95% CI: −3.53 to 7.20; t = 0.671; *p* = 0.521).

The sensitivity analyses performed after 1 min post-procedure demonstrated that the overall pooled effect was robust to the exclusion of individual studies. The primary analysis showed a small, non-significant effect favoring EBM compared with sweet solutions (SMD = 0.40; 95% CI −0.11 to 0.92), with high heterogeneity (I^2^ = 84.5%) and a 95% prediction interval ranging from −1.16 to 1.96, indicating that the true effect in a future comparable study could favor either intervention. Across sensitivity analyses, the exclusion of individual studies resulted in pooled estimates that remained stable (SMD range: 0.25 to 0.53), with no relevant changes in the direction or magnitude of the effect. The prediction intervals consistently crossed the null value, even when the exclusion of Velumula et al. [89] yielded a statistically significant pooled estimate (SMD = 0.53; 95% CI 0.03 to 1.03), indicating persistent uncertainty regarding the expected effect in future comparable studies. A reduction in heterogeneity to the moderate-to-high range was observed when excluding and Shanthi et al. [87] (I^2^ = 76.2%) and Velumula et al. [89] (I^2^ = 78.3%), suggesting that these studies contributed more prominently to between-study variability. Overall, the sensitivity analyses confirm that the meta-analytic results are robust, while the consistently high heterogeneity and prediction intervals spanning the null value indicate persistent between-study differences that are not explained by the influence of any single study (Table 4).

### 3.6. Effect of Expressed Breast Milk Versus Sweet Solutions on Neonatal Pain After 2 Minutes Post-Procedure

The meta-analysis of neonatal pain after 2 min post-procedure included five randomized controlled trials comparing EBM versus sweet solutions. The pooled analysis showed a small and non-significant effect favoring EBM compared with sweet solutions (SMD = 0.27; 95% CI −0.14 to 0.69; *p* = 0.14). The 95% prediction interval ranged from −0.46 to 1.00, indicating that the true effect in a future comparable study could favor either intervention. The confidence interval crossed the null value, indicating no statistically significant difference between groups at this time point. Heterogeneity was low and not statistically significant (I^2^ = 7.9%, *p* = 0.36), suggesting a high level of consistency in effect estimates across studies.

Subgroup analyses according to type of sweet solution also showed no statistically significant differences between EBM and glucose-based solutions (SMD = 0.25; 95% CI −3.84 to 4.34; I^2^ = 69.7%), sucrose (SMD = 0.05; 95% CI −1.21 to 1.31; I^2^ = 0%), or dextrose (SMD = 0.56; 95% CI −0.20 to 1.33). In all cases, the confidence intervals crossed the null value, indicating imprecision and uncertainty in the subgroup-specific estimates.

**Figure 7:**
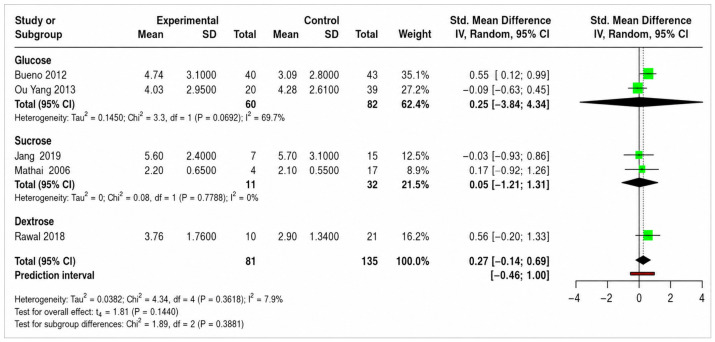
Effect of Expressed Breast Milk versus Sweet Solutions on Neonatal Pain After 2 Minutes Post-procedure in the studies included [75,77,79,83,84].

Importantly, no statistically significant differences were observed between subgroups (χ^2^ = 1.89, *p* = 0.38), indicating that the type of sweet solution did not significantly modify the effect of EBM on neonatal pain at 2 min post-procedure. Overall, the available evidence does not demonstrate a statistically significant difference between expressed breast milk and sweet solutions for reducing neonatal procedural pain after 2 min post-procedure (Figure 7).

The primary meta-analysis at 2 min post-procedure showed a small, non-significant overall effect (SMD = 0.27; 95% CI −0.14 to 0.69) with low heterogeneity (I^2^ = 7.9%) and a 95% prediction interval ranging from −0.46 to 1.00, indicating that the true effect in a future comparable study could favor either EBM or sweet solutions. Sensitivity analyses were performed using a leave-one-out approach to assess the robustness of the pooled estimate and the influence of individual studies on both effect size and between-study variability (Table 5). Across the leave-one-out analyses, pooled effect estimates remained stable, and the prediction intervals consistently crossed the null value, indicating persistent uncertainty regarding the expected effect in future comparable studies. Although exclusion of Ou Yang et al. [83] resulted in a statistically significant pooled effect (SMD = 0.44; 95% CI 0.03 to 0.84), the prediction interval (−0.10 to 0.98) continued to include the null value, suggesting that this finding should be interpreted with caution. Overall, these sensitivity analyses support the robustness of the primary findings and indicate that no single study materially influenced the overall conclusions.

### 3.7. GRADE Certainty of Evidence

Table 6 summarizes the certainty of the evidence for the three evaluated outcomes according to the GRADE approach. Overall, the certainty of evidence ranged from low to moderate. Low-certainty evidence was identified for neonatal pain during the procedure and 1 min after 1 min post-procedure, mainly due to substantial statistical heterogeneity and imprecision. In contrast, moderate-certainty evidence was observed for neonatal pain after 2 min post-procedure, with downgrading only for imprecision, while no serious concerns were identified regarding risk of bias, indirectness, inconsistency, or publication bias.

## 4. Discussion

This systematic review and meta-analysis synthesized evidence from 17 randomized controlled trials evaluating the efficacy of EBM compared with commonly used sweet solutions for neonatal procedural pain. Across the evaluated time points (during the procedure, 1 min, and 2 min post-procedure), the pooled analyses consistently demonstrated no statistically significant differences between EBM and sweet solutions. Although the magnitude and direction of the pooled effects varied across studies and comparators, none of the overall analyses or subgroup analyses according to sweet solutions reached statistical significance. Furthermore, considerable heterogeneity was observed during the procedure and at 1 min post-procedure, whereas heterogeneity was low at 2 min, indicating that the consistency of treatment effects differed according to the timing of pain assessment.

From an interpretative perspective, these findings suggest that EBM provides an analgesic effect broadly comparable to that of commonly used sweet solutions for neonatal procedural pain. However, the substantial heterogeneity observed in the analyses performed during the procedure and after 1 min post-procedure indicates that the relative effectiveness of EBM is likely influenced by differences in study populations, procedural characteristics, comparator solutions, and methodological approaches [91,92]. Consequently, the pooled estimates should be interpreted as average effects across heterogeneous clinical settings rather than evidence supporting the superiority of either intervention. The consistently non-significant findings across the primary analyses, subgroup analyses, and sensitivity analyses further suggest that the currently available evidence does not demonstrate a clinically meaningful advantage of one intervention over the other.

From a clinical perspective, these findings indicate that EBM may represent an appropriate non-pharmacological alternative to sweet solutions for reducing procedural pain in neonates. Importantly, no statistically significant differences were identified when EBM was compared with glucose-, sucrose-, or dextrose-based solutions, and interaction tests did not demonstrate significant differences between comparator subgroups at any evaluated time point. Although the point estimates for dextrose were consistently larger than those observed for glucose or sucrose, these differences were accompanied by wide confidence intervals, substantial heterogeneity, and non-significant subgroup interaction tests. Therefore, the available evidence does not support the superiority of any specific sweet solution over EBM, and the choice of intervention should also consider factors such as local clinical protocols, breastfeeding promotion, availability of sweet solutions, and parental preferences [19,93].

The larger effect estimates observed in studies comparing EBM with dextrose nevertheless deserve consideration. Hyperosmolar glucose solutions such as dextrose are thought to produce stronger activation of sweet taste receptors than breast milk because of their substantially higher carbohydrate concentration [94,95]. Experimental evidence suggests that activation of oral sweet taste receptors rapidly stimulates endogenous opioid pathways and other descending inhibitory mechanisms, producing immediate analgesic effects during brief painful procedures [96,97]. In contrast, although EBM contains lactose together with numerous bioactive compounds with potential analgesic properties, its lower carbohydrate concentration and natural variability in composition between mothers may contribute to greater variability in analgesic response [98,99]. Differences in administered volume, timing of administration, gestational maturity, feeding status, and procedural characteristics may further explain the variability observed across individual studies. Consequently, although biological plausibility exists for stronger analgesic effects with concentrated sweet solutions, the present meta-analysis did not demonstrate statistically significant differences supporting the superiority of dextrose over EBM [98,99].

The pattern of heterogeneity observed across analyses provides additional insight into the interpretation of these findings. Considerable heterogeneity was identified during the procedure and at 1 min post-procedure, whereas heterogeneity was low after 2 min post-procedure. This suggests that variability in treatment effects was more pronounced during the immediate response to painful stimulation than during later pain assessments. Several factors may account for this variability, including differences in comparator solutions (glucose, sucrose, and dextrose), gestational age, birth weight, type of painful procedure, timing of intervention administration, administered volumes, pain assessment instruments, and outcome assessment schedules [100,101,102]. These sources of clinical and methodological diversity are likely to influence both the magnitude and consistency of analgesic responses. Although subgroup analyses according to the type of sweet solution did not demonstrate statistically significant interaction effects at any evaluated time point, these findings should be interpreted cautiously because the number of studies within individual subgroups was limited, resulting in wide confidence intervals and reduced statistical power to detect subgroup differences.

The leave-one-out sensitivity analyses further strengthened the robustness of the primary findings. Sequential exclusion of individual studies did not materially alter the magnitude, direction, or statistical significance of the pooled estimates across any of the evaluated time points. During the procedure, exclusion of Kazmi et al. [78] substantially reduced heterogeneity while leaving the pooled effect essentially unchanged, suggesting that this study contributed importantly to between-study variability but did not influence the overall conclusion. Similarly, after 1 min, exclusion of Velumula et al. [89] or Shanthi et al. [87] moderately reduced heterogeneity without changing the non-significant pooled estimate, whereas the analyses performed after 2 min post-procedure remained highly consistent regardless of the study excluded. Collectively, these findings indicate that the overall conclusions are robust and are not driven by the influence of any single trial.

Visual inspection of the funnel plots suggested mild asymmetry for the analyses performed during the procedure and after 1 min post-procedure. However, this apparent asymmetry coincided with substantial between-study heterogeneity and the presence of influential studies located at opposite extremes of the distribution, particularly Rawal et al. [84], Velumula et al. [89], and Shanthi et al. [87]. Consequently, the observed asymmetry is more plausibly explained by genuine between-study variability and differences in effect magnitude than by publication bias alone [28]. Moreover, the relatively small number of included studies in each meta-analysis limits the reliability of funnel plot interpretation and precludes firm conclusions regarding the presence of small-study effects.

From a mechanistic perspective, the analgesic effects of EBM are biologically plausible and likely result from multiple complementary mechanisms rather than a single pharmacological pathway. Breast milk contains lactose, tryptophan, endogenous opioid-like peptides, melatonin, oxytocin, and numerous bioactive compounds that may contribute to modulation of nociceptive processing through neurobehavioral regulation and autonomic stabilization [59,103]. In addition, the administration of EBM is frequently accompanied by familiar olfactory, gustatory, and tactile stimuli that may promote vagal activation, behavioral organization, and improved physiological regulation during painful procedures [59,104]. These multimodal sensory inputs may collectively attenuate pain responses even if their immediate analgesic effect is less pronounced than that produced by highly concentrated sweet solutions [50,105].

Beyond these immediate neurophysiological mechanisms, breast milk also contains immunomodulatory, anti-inflammatory, antioxidant, and neurotrophic components that support neonatal physiological stability and neurodevelopment [106]. Although these biological properties are unlikely to produce rapid analgesia during brief procedures, they may contribute to improved stress regulation and developmental adaptation over time [107]. Therefore, the potential benefits of EBM should not be evaluated solely according to immediate pain scores but also within the broader context of neonatal supportive care. This perspective reinforces the concept that EBM represents a physiologically appropriate intervention with potential benefits extending beyond procedural analgesia alone [108].

In this context, EBM should not be interpreted as either superior or inferior to sweet solutions based on the current evidence. Rather, the findings of this review support its role as a safe, feasible, and developmentally appropriate component of multimodal non-pharmacological pain management strategies [38,87]. Given the absence of statistically significant differences between interventions, clinicians may reasonably consider EBM as an alternative when sweet solutions are unavailable, contraindicated, or when promoting breastfeeding and maternal–infant bonding is a clinical priority. Likewise, sweet solutions remain appropriate evidence-based interventions for minor procedural pain and may be selected according to local protocols, resource availability, and individual patient circumstances [94,109].

From a clinical perspective, these findings are consistent with contemporary recommendations advocating multimodal approaches for neonatal procedural pain management. Current evidence-based guidelines recommend combining effective non-pharmacological interventions—including oral sweet solutions, non-nutritive sucking, skin-to-skin care, breastfeeding or expressed breast milk whenever feasible, facilitated tucking, and other supportive measures—to minimize procedural pain in neonatal intensive care settings [110,111,112]. Rather than supporting replacement of one intervention by another, the present findings reinforce the importance of integrating EBM into individualized, multimodal pain management strategies tailored to the clinical context and the needs of each infant.

This review has several methodological strengths. It was conducted in accordance with PRISMA guidelines and prospectively registered in PROSPERO. Only randomized controlled trials were included, reducing the risk of confounding associated with non-randomized designs [24]. A comprehensive search strategy across multiple electronic databases was performed, and study selection, data extraction, and risk of bias assessment were conducted using standardized methods [24]. The use of random-effects models with Hedges’ g correction appropriately accounted for between-study variability, while predefined subgroup analyses according to comparator type and timing of pain assessment improved clinical interpretability of the findings [23,24]. In addition, leave-one-out sensitivity analyses demonstrated that the pooled estimates were robust to the exclusion of individual studies, and structured assessment of methodological quality using the Cochrane RoB 2 tool further strengthened the overall credibility of the evidence synthesis [21,24,113,114,115].

Several limitations should also be considered when interpreting these findings. First, considerable heterogeneity was observed in the analyses performed during the procedure and at 1 min post-procedure, reducing confidence in those pooled estimates despite the stability demonstrated in sensitivity analyses [116]. Although heterogeneity was low at 2 min, that analysis included fewer studies, limiting statistical power and the precision of subgroup comparisons. Second, important clinical variability existed across trials with respect to gestational age, type of painful procedure, comparator solution, concentration and volume of sweet solutions, timing of intervention administration, and outcome assessment schedules. Although pain scores were standardized using standardized mean differences, variability in the pain assessment instruments may still have introduced residual measurement inconsistency because these scales differ in their psychometric properties and construct definitions [27]. Furthermore, several subgroup analyses included a limited number of studies, resulting in wide confidence intervals and reducing the ability to detect true differences between comparator solutions. Finally, the mild asymmetry observed in the funnel plots should be interpreted cautiously because the relatively small number of studies and the substantial heterogeneity observed in some analyses reduce the reliability of visual assessment of publication bias [117].

Future research should focus on improving the methodological consistency of randomized controlled trials evaluating non-pharmacological interventions for neonatal procedural pain. Greater standardization of pain assessment instruments, timing of outcome measurement, intervention protocols, and methods of EBM administration would facilitate comparisons across studies and reduce clinical heterogeneity. Individual participant data meta-analyses may further clarify whether specific neonatal subgroups, defined by gestational age, birth weight, feeding status, or clinical condition, derive greater benefit from EBM than from sweet solutions. In addition, future randomized trials should evaluate whether combining EBM with other evidence-based non-pharmacological interventions, such as non-nutritive sucking, skin-to-skin care, facilitated tucking, or maternal voice, provides additive analgesic effects beyond those achieved by either intervention alone. Finally, studies evaluating repeated painful procedures, dose-response relationships, optimal timing of administration, and longer-term neurodevelopmental outcomes would help clarify the broader role of expressed breast milk within multimodal neonatal pain management strategies.

Overall, the current evidence indicates that expressed breast milk and commonly used sweet solutions provide comparable analgesic effects for neonatal procedural pain, with no statistically significant differences identified during the procedure or at 1 or 2 min post-procedure. Although considerable heterogeneity was present in some analyses, the consistency of the sensitivity analyses supports the robustness of these findings. Consequently, expressed breast milk should be considered a safe and clinically appropriate option within multimodal neonatal pain management, particularly in settings where breastfeeding promotion, maternal–infant bonding, and family-centred care are prioritized. Further high-quality randomized controlled trials using standardized methodologies are needed to improve the certainty of the evidence and determine whether specific neonatal populations may derive greater benefit from one intervention over another.

## 5. Conclusions

The present systematic review and meta-analysis found no statistically significant differences between EBM and sweet solutions for reducing neonatal procedural pain during the procedure or at 1 and 2 min post-procedure. Although the pooled effect estimates generally favored EBM, all confidence intervals crossed the null value, and substantial heterogeneity was observed during the procedure and at 1 min post-procedure, whereas heterogeneity was low at 2 min. Subgroup analyses according to the type of sweet solution (glucose, sucrose, and dextrose) also showed no statistically significant differences, indicating that the comparator did not significantly modify the treatment effect.

These findings support expressed breast milk as a biologically plausible, safe, and clinically appropriate non-pharmacological option for neonatal pain management. While the available evidence does not demonstrate superiority of EBM over conventional sweet solutions, it suggests that EBM provides comparable analgesic effects and may be considered as part of multimodal pain management strategies, particularly in settings where breastfeeding promotion and mother–infant bonding are priorities. Nevertheless, the substantial clinical and methodological heterogeneity across studies limits the certainty of the evidence. Further high-quality randomized controlled trials using standardized intervention protocols, pain assessment methods, and outcome measurement time points are required to clarify the comparative effectiveness of EBM and sweet solutions in neonatal procedural pain management.

## Figures and Tables

**Table 1:** Search strategy for each database.

Database	Electronic Search
Medline/PubMed	(“Breast Milk”[Mesh] OR “breast milk” OR “expressed breast milk” OR “mother’s milk”) AND (“Infant, Newborn”[Mesh] OR neonate OR newborn) AND (“Pain”[Mesh] OR pain OR analgesia OR “procedural pain”)
Scopus	TITLE-ABS-KEY (“expressed breast milk” OR “breast milk” OR “mother* milk”) AND TITLE-ABS-KEY (neonat* OR newborn OR “new born” OR “infant, newborn”) AND TITLE-ABS-KEY (pain OR analges* OR “procedural pain” OR “pain relief”)
CINAHL	(“breast milk” OR “expressed breast milk” OR “mother’s milk”) AND (neonate OR newborn OR neonat*) AND (pain OR analgesia OR “procedural pain”)
Cochrane Library	(“breast milk” OR “expressed breast milk” OR “mother’s milk”) AND (neonate OR newborn OR neonat*) AND (pain OR analgesia OR “procedural pain”)
Google scholar	“breast milk” neonate pain procedural pain “expressed breast milk” neonate analgesia “breast milk” heel lance neonate

**Table 2:** Characteristics of the studies analyzed.

Study	Country	Population	Procedure	Pain Assessment Time Points	Sweetener Comparison	Outcome of Pain	Study Design
Bembich et al. 2018 [74].	Italy	80 neonates, 37–42 weeks	Heel lance	During procedure	Glucose 2 mL (20%)	NIPS	Parallel RCT
Bueno et al. 2012 [75].	Brazil	83 neonates, 34–36 weeks	Heel lance	Postprocedure 0.5–1–1.5–2–2.5–3 min	Glucose 2 mL (25%)	PIPP	Parallel RCT
Collados-Gómez et al. 2018 [76].	Spain	66 neonates, <37 weeks	Venipuncture	Postprocedure: 0.5 min	Sucrose 2 mL (24%)	PIPP	Parallel RCT
Jang et al. 2019 [77].	South Korea	45 neonates, <35 weeks	ROP screening	During procedure; postprocedure 2 min	Sucrose (24%)	PIPP	Parallel RCT
Kazmi et al. 2020 [78].	Pakistan	400 neonates, 34–37 weeks	Venipuncture	During procedure	Dextrose 25%	PIPP	Parallel RCT
Mathai et al. 2006 [79].	India	104 neonates, >37 weeks	Heel lance	Postprocedure 0.5–1–2–4 min	Sucrose 2 mL (20%)	DAN	Parallel RCT
Mirlashari et al. 2021 [80].	Iran	63 neonates, ≤32 weeks	ROP screening	During procedure; postprocedure; 5–10–15 min	Sucrose 24% 0.5 mL/kg	ALPS-Neo	Parallel RCT
Modaresi et al. 2024 [81].	Iran	99 neonates, ≥37 weeks	Venipuncture	During procedure; postprocedure: 0.5–1 min	Sucrose 24% 2 mL	NIAPAS	Parallel RCT
Nayak et al. 2020 [82].	India	45 neonates, approximately 32 weeks	ROP screening	Preprocedure: 1 min	Dextrose 10%	PIPP	Parallel RCT
Ou-Yang et al. 2013 [83].	Taiwan	123 neonates, <37 weeks	Heel lance	Postprocedure: 1–2–3 min	Glucose 25% 5 mL	N-PASS	Parallel RCT
Rawal et al. 2018 [84].	India	33 neonates, 34–36 + 6 weeks	Heel lance	Postprocedure: 0 to 0.5–0.5 to 1 1 to 1.5–2 to 2.5 min	Dextrose 25% 2 mL	PIPP	Parallel RCT
Rodrigues et al. 2017 [85].	India	40 neonates, <37 weeks	Nasopharyngeal suctioning	During procedure; Postprocedure; 1–5 min	Dextrose 25% 0.3 mL/kg	PIPP	Parallel RCT
Şener Taplak et al. 2017 [86].	Turkey	60 neonates, ≤32 weeks	ROP screening	Preprocedure: 5 min; during procedure; postprocedure: 5 min	Sucrose 33% 1 mL	PIPP	Parallel RCT
Shanthi et al. 2024 [87].	India	72 neonates, ≥34 to ≤42 weeks	Heel lance	During procedure: 0 min; postprocedure: 0.5 and 1 min	Dextrose 10% 1 mL	PIPP-R	Parallel RCT
Turan et al. 2021 [88].	Turkey	51 neonates, <32 weeks	ROP screening	Preprocedure: 0 min; during procedure: 0.5, 1, and 1.5 min; postprocedure: 0.5 min	Sucrose 24% 0.3 mL	NIPS	Parallel RCT
Velumula et al. 2022 [89].	United States	88 neonates, 30 + 1 to 36 + 6 weeks	Heel lance	Preprocedure: 0 min; during procedure; postprocedure: 0.5, 1, 1.5, and 2 min	Sucrose 24% 0.5 mL	PIPP-R	Parallel RCT
Yilmaz et al. 2011 [90].	Turkey	120 neonates, 37–42 weeks	Heel lance	Preprocedure; during procedure; postprocedure	Sucrose 20% 2 mL	NIPS	Parallel RCT

NIPS: Neonatal Infant Pain Scale, PIPP: Premature Infant Pain Profile, PIPP-R: Premature Infant Pain Profile-Revised, N-PASS: Neonatal Pain Agitation and Sedation Scale, DAN: Douleur Aiguë du Nouveau-né, ALPS-Neo: Acute Neonatal Pain Assessment Scale, NIAPAS: Neonatal Infant Acute Pain Assessment Scale, RCT: Randomized Controlled Trial, ROP: Retinopathy Of Prematurity.

**Table 3:** Sensitivity analyses during the procedure.

Analysis	Excluded Study	k	SMD (95% CI)	Prediction Interval	I^2^
Primary analysis	None	11	0.15 (−0.18 to 0.47)	−0.89 to 1.18	82.6%
Leave-one-out analysis	Bembich et al. [74].	10	0.16 (−0.20 to 0.53)	−0.94 to 1.26	83.4%
	Jang et al. [77].	10	0.16 (−0.21 to 0.52)	−0.93 to 1.24	83.9%
	Kazmi et al. [78].	10	0.01 (−0.25 to 0.26)	−0.52 to 0.53	16.6%
	Mirlashari et al. [80].	10	0.17 (−0.20 to 0.53)	−0.92 to 1.25	83.6%
	Modaresi et a [81].	10	0.09 (−0.26 to 0.45)	−0.99 to 1.18	84.3%
	Nayak et al. [82].	10	0.11 (−0.24 to 0.46)	−0.97 to 1.19	84.3%
	Rodrigues et al. [85].	10	0.15 (−0.21 to 0.52)	−0.95 to 1.25	83.9%
	Sener Taplak et al. [86].	10	0.14 (−0.23 to 0.50)	−0.97 to 1.24	84.2%
	Turan et al. [88].	10	0.20 (−0.14 to 0.54)	−0.82 to 1.23	81.9%
	Velumula et al. [89].	10	0.19 (−0.16 to 0.55)	−0.87 to 1.26	79.7%
	Yilmaz et al. [90].	10	0.19 (−0.15 to 0.54)	−0.85 to 1.24	82.5%
Excluding high-RoB studies	Modaresi et al. [81]. Yilmaz et al. [90].	9	0.14 (−0.24 to 0.52)	−0.97 to 1.26	84.5%

**Table 4:** Sensitivity Analyses After 1 Minute Post-procedure.

Analysis	Excluded Study	k	SMD (95% CI)	Prediction Interval	I^2^
Primary analysis	None	10	0.40 (−0.11 to 0.92)	−1.16 to 1.96	84.5%
Leave-one-out analysis	Bueno et al. [75].	9	0.36 (−0.22 to 0.94)	−1.32 to 2.05	85.2%
	Collados-Gomez et al. [76].	9	0.44 (−0.15 to 1.02)	−1.28 to 2.15	86%
	Mathai et al. [79].	9	0.37 (−0.19 to 0.94)	−1.28 to 2.03	86%
	Modaresi et al. [81].	9	0.38 (−0.20 to 0.96)	−1.33 to 2.08	85.8%
	Ou Yang et al. [83].	9	0.46 (−0.10 to 1.03)	−1.19 to 2.12	85.6%
	Rawal et al. [84].	9	0.32 (−0.21 to 0.84)	−1.23 to 1.86	84.7%
	Rodrigues et al. [85].	9	0.46 (−0.10 to 1.02)	−1.18 to 2.11	85.8%
	Shanthi et al. [87].	9	0.25 (−0.21 to 0.71)	−1 to 1.50	76.2%
	Turan et al. [88].	9	0.47 (−0.09 to 1.03)	−1.17 to 2.11	85.7%
	Velumula et al. [89].	9	0.53 (0.03 to 1.03)	−0.87 to 1.93	78.3%
Excluding high-RoB studies	Modaresi et al. [81]. Rawal et al. [84].	8	0.27 (−0.33 to 0.88)	−1.42 to 1.97	86%

**Table 5:** Sensitivity Analyses After 2 Minutes Post-procedure.

Analysis	Excluded Study	k	SMD (95% CI)	Prediction Interval	I^2^
Primary analysis	None	5	0.27 (−0.14 to 0.69)	−0.46 to 1	7.9%
Leave-one-out analysis	Bueno et al. [75].	4	0.10 (−0.39 to 0.60)	−0.50 to 0.71	0%
	Jang et al. [77].	4	0.31 (−0.23 to 0.86)	−0.63 to 1.25	20.6%
	Mathai et al. [79].	4	0.28 (−0.30 to 0.85)	−0.68 to 1.24	30.1%
	Ou Yang et al. [83].	4	0.44 (0.03 to 0.84)	−0.10 to 0.98	0%
Excluding high-RoB studies	Rawal et al. [84].	4	0.21 (−0.33 to 0.75)	−0.80 to 1.22	20.6%

**Table 6:** Summary of effect and certainty of evidence estimators for outcome.

Outcomes	Studies	Participants	Effect Estimate (SMD, 95% CI)	GRADE	Justification
Neonatal pain during the procedure	11	804	0.15 (−0.18 to 0.47)	Low	The certainty of evidence was rated as low. No downgrading was applied for risk of bias, as most studies were judged to be at low risk of bias (7/11), despite two studies presenting some concerns and two at high risk of bias; sensitivity analyses excluding the high-risk studies did not materially change the pooled effect estimate. The certainty was downgraded to one level for inconsistency because considerable statistical heterogeneity was observed (I^2^ = 82.6%) and one level for imprecision because the 95% confidence interval crossed the line of no effect. No downgrading was applied for indirectness, as the included studies directly addressed the review question. Although Egger’s regression test suggested funnel plot asymmetry, no downgrading was applied for publication bias because the observed asymmetry was considered more likely to reflect substantial between-study heterogeneity and the influence of a small number of outlying studies rather than publication bias, consistent with the visual inspection of the funnel plot.
Neonatal pain After 1 Minute Post-procedure.	10	618	0.40 (−0.11 to 0.92)	Low	The certainty of evidence was rated as low. No downgrading was applied for risk of bias, as most studies were judged to be at low risk of bias (6/10), despite two studies presenting some concerns and two at high risk of bias; sensitivity analyses excluding the high-risk studies did not materially change the pooled effect estimate. The certainty was downgraded to one level for inconsistency because considerable statistical heterogeneity was observed (I^2^ = 84.5%) and one level for imprecision because the 95% confidence interval crossed the line of no effect. No downgrading was applied for indirectness, as the included studies directly addressed the review question. No downgrading was applied for publication bias because visual inspection of the funnel plot did not suggest substantial publication bias, and Egger’s regression test did not detect significant funnel plot asymmetry
Neonatal pain After 2 Minutes Post-procedure.	5	216	0.27 (−0.14 to 0.69)	Moderate	The certainty of evidence was rated as moderate. No downgrading was applied for risk of bias, as most studies were judged to be at low risk of bias (3/5), with one study presenting some concerns and one study at high risk of bias; moreover, sensitivity analysis excluding the high-risk study did not materially change the pooled effect estimate. No downgrading was applied for inconsistency due to the low statistical heterogeneity (I^2^ = 7.9%) or for indirectness, as the included studies directly addressed the review question regarding the population, intervention, comparator, and outcome. The certainty of evidence was downgraded by one level for imprecision because only five studies were included and the 95% confidence interval crossed the line of no effect. Publication bias could not be formally assessed because fewer than 10 studies were available; therefore, no downgrading was applied for this domain.

## Data Availability

No new data were created or analyzed in this study. Data sharing is not applicable to this article.

## Supplementary Materials

The following supporting information can be downloaded at: https://www.mdpi.com/article/10.3390/biomedicines14071565/s1.

## Abbreviations

The following abbreviations are used in this manuscript:

EBM	Expressed Breast Milk
REML	Restricted Maximum Likelihood
IASP	Association for the Study of Pain
RCT	Randomized Controlled Trial
RoB2	Risk of Bias 2
PRISMA	Preferred Reporting Items for Systematic Reviews and Meta-Analyses
NICUs	Neonatal Intensive Care Units
ROP	Retinopathy of Prematurity
PIPP	Premature Infant Pain Profile
PIPP-R	Premature Infant Pain Profile-Revised
NIPS	Neonatal Infant Pain Scale
NIAPAS	Neonatal Infant Acute Pain Assessment Scale
DAN	Douleur Aiguë du Nouveau-né
N-PASS	Neonatal Pain, Agitation and Sedation Scale
ALPS-Neo	Acute Neonatal Pain Assessment Scale
SMD	Standardized Mean Difference
CI	Confidence Interval
